# Comorbid status and the faecal microbial transplantation failure in treatment of recurrent *Clostridioides difficile* infection – pilot prospective observational cohort study

**DOI:** 10.1186/s12879-020-4773-x

**Published:** 2020-01-16

**Authors:** M. Kachlíková, P. Sabaka, A. Koščálová, M. Bendžala, Z. Dovalová, I. Stankovič

**Affiliations:** 1 Department of Infectology and Geographical Medicine, Faculty of Medicine, Comenius University in Bratislava, Bratislava, Slovak Republic; 2 Department of Infectology and Geographical Medicine, Faculty of Medicine, Slovak Medical University, Bratislava, Slovak Republic

**Keywords:** *Clostridioides difficile* infection, Faecal microbial transplantation, Comorbidity

## Abstract

**Background:**

Faecal microbial transplantation (FMT) is currently the most effective treatment of recurrent *Clostridioides difficile* infection (CDI). However, up to 20% of patients experience further recurrences after single FMT. The mechanisms that lead to FMT failure and its risk factors are poorly understood. Comorbidity is one of the risk factors of the failure of standard antibiotic therapy of recurrent CDI. It is not known if comorbidity is also associated with the risk of FMT failure.

**Methods:**

We conducted a prospective observational cohort study in order to elucidate if comorbid status is associated with FMT failure. Patients with microbiologically proven recurrent CDI were recruited and underwent FMT via retention enema. Patients were followed up for 12 weeks after FMT for signs and symptoms of CDI recurrence. Single FMT failure was defined as recurrence of diarrhoea and a positive stool test for the presence of *C. difficile* antigen or toxin at any time point during the 12 weeks of follow-up. We assessed the association of single FMT failure with possible manageable and unmanageable risk factors. As a surrogate of comorbid status, we used Charlson Comorbidity Index (CCI) ≥ 7.

**Results:**

A total of 60 patients that underwent single FMT (34 women, 26 men) were included in the study. Overall, 15 patients (25%) experienced single FMT failure. 24 patients (40%) had CCI ≥ 7, and 45.0% patients with CCI ≥ 7 experienced failure of single FMT. Patients who experienced single FMT failure had a significantly higher CCI and significantly lower albumin concentration as compared to patients who experienced single FMT success. There was no difference in age, C-reactive protein concentration, leukocyte count and time from FMT to first defecation. In multivariate analysis, CCI ≥ 7 was positively associated with the failure of single FMT. Analysis was controlled for sex, age, time from FMT to first defecation, concomitant PPI therapy, severe CDI, hospital-acquired infection and albumin concentration.

**Conclusions:**

Comorbid status surrogated by CCI is positively associated with the failure of single FMT in the treatment of recurrent CDI.

## Background

*Clostridioides difficile,* formerly known as *Closridium difficle* is an anaerobic, spore-forming, Gram-positive bacillus. It may be part of normal intestinal flora in neonates but can also lead to severe health care-associated colitis. Clinical spectrum of diseases caused by *Clostridioides difficile* infection (CDI) ranges from mild diarrhea to severe pseudomembranous colitis with toxic megacolon and ileus. Low diversity of intestinal microbiome allowing intestinal overgrowth of *C. difficile* is the most important risk factor for developing CDI [1–6]. CDI is the most common cause of health care-associated diarrhea and one of the most common causes of all health care-associated infections in Western countries [3, 4]. The occurrence of CDI in hospitalized patients greatly increases mortality and overall cost of the treatment [5, 6]. A significant portion of the medical and economic burdens of CDI is caused by recurrent CDI [6]. More than 20% of patients with CDI experience the recurrence of disease within 2 months, and if CDI recurs, the chance for a subsequent recurrence increases to 60% [7]. The probability of developing recurrent CDI is greatly affected by the presence of severe underlying diseases, such as chronic heart failure, diabetes mellitus or other serious comorbid conditions [8–12]. Therefore, the treatment of recurrent CDI is very challenging especially in older comorbid patients [13]. Faecal microbial transplantation (FMT) is currently the most effective treatment for recurrent CDI [14, 15]. According to a large meta-analysis, it prevents further recurrences of CDI in more than 90% of patients in an unselected population, and the efficacy of FMT varies from 80 to 95% between studies [15]. Its efficacy is based on its ability to restore the physiologic diversity of the faecal microbiome, which is believed to be essential in prevention of further recurrences [16]. However, even FMT fails to prevent further recurrences in 5 to 20% of patients [15]. The mechanisms of FMT failure are poorly understood. Some potential predictors of FMT failure were identified, but the association of FMT failure with most of the plausible causes remained unknown. Only 3 studies [17–19]. Since severe underlying diseases are predictors of recurrent CDI, we suppose that it may also contribute to the risk of FMT failure. Therefore, we conducted a prospective observational study in order to elucidate if comorbidity is associated with FMT failure. To objectively assess comorbid status, we used the Charlson Comorbidity Index (CCI) as a well-established surrogate marker of comorbidity. It was developed and validated as a measure of 1-year mortality risk and a marker of disease burden. The CCI is used extensively in clinical research to address the influence of comorbidities on prognosis and outcome [20, 21].

## Methods

### Design

We conducted a prospective observational cohort study in order to elucidate if comorbidity expressed by the CCI is associated with FMT failure.

### Patients

We recruited patients admitted with the recurrent CDI to the Department of Infectology and Geographical Diseases of University Hospital in Bratislava from January 2017 to August 2019. The inclusion criterion was the presence of recurrent CDI. Recurrent CDI was defined as recurrent diarrhoea with positivity for *C. difficile* toxin in stool within 8 weeks after the previous episode of CDI treated by standard antibiotic therapy (vancomycin 125 mg orally every 6 h for at least 10 days). Exclusion criteria were pregnancy, alcohol abuse, illicit drug abuse, severe dementia, paraplegia, quadriplegia, concomitant antibiotic therapy and FMT within the past 12 weeks. The patients with suspected recurrent CDI were screened for exclusion and inclusion criteria at the time of admission. The medical history was obtained, and blood from the cubital vein (for biochemical examination of C-reactive protein concentration, albumin concentration and blood count) was drawn by a certified nurse. Age and presence of comorbidities found in the medical history were used to calculate the CCI using macros in MS Excel MS Office 2010 (Microsoft Corporation, Redmond, Washington, USA), as proposed by Hall et al. [22]. A specimen of fresh stool was obtained to examine for the presence of *C. difficile* toxin and antigen. The patients with diarrhea with positive *C. difficile* toxin and antigen using enzyme-linked immunosorbent assay were diagnosed with CDI according to European Society of Clinical Microbiology and Infectious Diseases guidelines [23]. Patients who fulfilled inclusion and exclusion criteria were recruited. All patients signed informed consent. After recruitment, the patients were treated with vancomycin 125 mg orally every 6 h for 10 days. Antibiotic therapy was discontinued 48 h before the FMT. After this washout period, the FMT was performed. Patients were then observed for 24 h for possible complications and discharged.

### Donors

Healthy volunteers less than 50 years of age were screened through a specific questionnaire about possible risk factors for potentially transmittable diseases. Exclusion criteria for donors were intake of antibiotics in the previous 3 months, lifestyle habits associated with increased risk for contracting infections in the last 6 months (travel to tropical areas, unprotected sexual intercourse with new partner, tattoo, piercing, surgery, endoscopic examination, and transfusion of blood and blood products), diarrhoea or other gastrointestinal symptoms in the last 3 months, history of inflammatory bowel disease, cancer, systemic rheumatic diseases, diabetes mellitus, or treatment with immunosuppressants. Potential donors underwent blood and stool examinations for potentially transmittable diseases. The blood samples were tested for hepatitis A, B, C and E, HIV, Epstein-Barr virus, cytomegalovirus, *Treponema pallidum, Entamoeba histolytica, Toxoplasma gondii, Toxocara canis, cati and suis*. We excluded donors tested positive for these pathogens. Also donors with antibodies against *Toxoplasma gondii* (indicating latent infection) or antibodies against *Entamoeba histolytica* and *Toxocara canis, cati and suis* (that might indicate past resolved infection) were excluded. We do so in order to prioritize safety of recipients before number of potential donors. The stool samples were tested for *C. difficile* antigen and toxin as well as *Helicobacter pylori, Giardia intestinalis* and *Entamoeba histolytica* antigens. Stool samples were also cultivated for the presence of *Salmonella* species, *Campylobacter jejuni* and multidrug resistant Gram-negative bacteria. Microscopic examination to exclude infections of the gastrointestinal tract with protozoa and helminths were performed from 3 independent stool samples. All donors were questioned for the presence of any recent acute gastrointestinal symptoms or use of new drugs or antibiotics directly before every donation.

### Faecal microbial transplantation procedure

Faeces were collected by the donor on the day of the FMT procedure and transported to our hospital without any unnecessary delay. A total of 30 mg of fresh faeces were diluted with 150 mL of 0.9% sterile saline solution. The solution was blended and homogenized and then filtered to remove any larger solid compounds. The homogenized and filtered solution was then poured into a sterile container. Within 6 h after the delivery of faeces by the donor, the solution was instilled into the patient intrarectally using an enema catheter. At the time of instillation, the patients were placed in the supine position and were asked to maintain this position for at least 2 h. After the procedure, patients were instructed to not defecate for at least 2 h. All FMT procedures were performed as inpatient procedures in our institution.

### Outcomes and follow-up

**Fig. 1:**
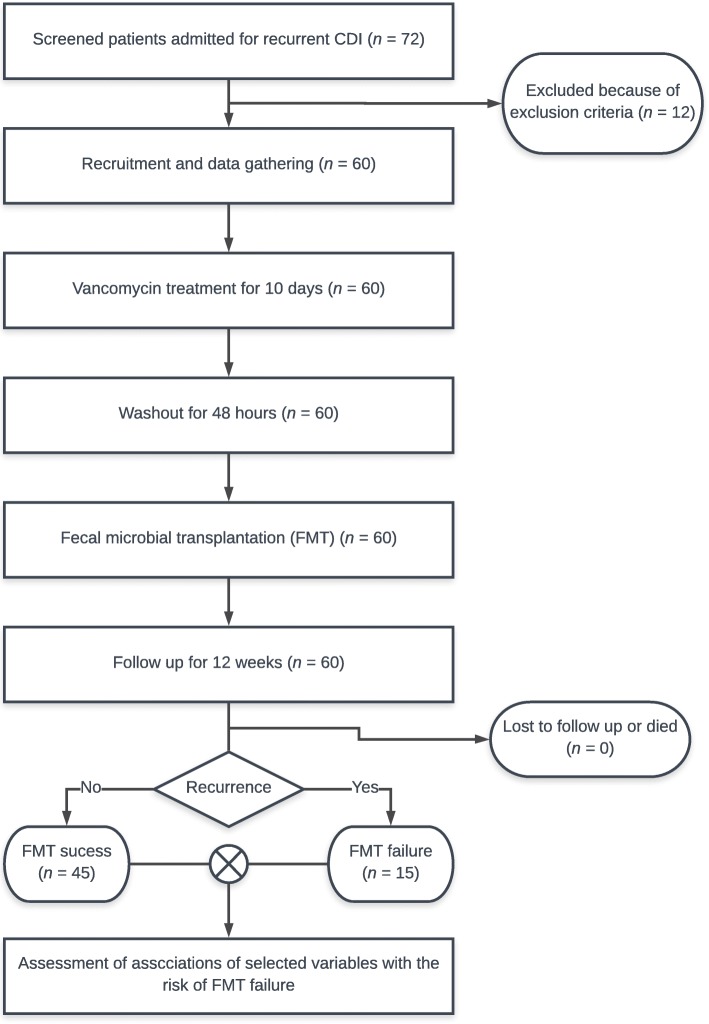
Study design flowchart. CDI – Clostridioides difficile infection, FMT – faecal microbial transplantation

The primary end point was the reappearance of diarrhoea (at least 3 watery or loose stools per day for at least 2 or more consecutive days) associated with CDI less than 12 weeks after the FMT procedure. Patients or relatives taking care of patients were instructed to keep stool diaries and were followed up telephonically. During routine weekly telephonic follow-up, patients were asked about symptoms of CDI recurrence (diarrhoea, bloating or abdominal pain) and frequency of stools. Also, patients were instructed to call or visit investigators in case of recurrence of diarrhoea, bloating or abdominal pain. In patients with recurrent symptoms, stool samples were examined for *C. difficile* antigen and toxin. Single FMT failure was defined as recurrence of diarrhoea (at least 3 watery or loose stools per day for at least 2 or more consecutive days) with a stool sample positive for *C. difficile* antigen and/or toxin within 12 weeks from the end of the therapy (Fig. 1).

**Fig. 2:**
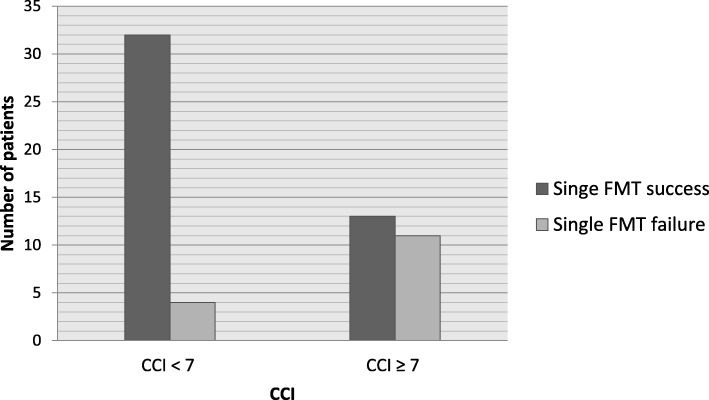
Number of patients with single FMT failure divided by cut-off CCI ≥ 7. CCI – Charlson Comorbidity Index, FMT – faecal microbial transplantation

### Laboratory assays

The presence of *C. difficle* GDH antigen and toxin were assessed using rapid-tests for rapid on-site diagnostics and later confirmed from fresh stool samples by enzyme-linked immunosorbent assay (ELISA). Stool rapid tests tests for *C. difficile* GDH antigen were performed using Rapid-Viditest *C. difficile* Ag (VIDIA spol. s r.o., Vestec, Czech Republic) and for *C. difficile* toxin were performed using immunochromatography RIDA®QUICK *Clostridium difficile* Toxin A/B (R-Biopharm AG, Pfungstadt, Germany). Confirmation ELISA tests for *C. difficle* GDH antigen were performed using RIDASCREEN® *Clostridium difficile* GDH (R-Biopharm AG, Pfungstadt, Germany) and for *C. difficile* toxins were performed using RIDASCREEN® *Clostridium difficile* Toxin A/B (R-Biopharm AG, Pfungstadt, Germany). All samples (positive or negative by rapid-tests) were retested using ELISA and results of ELISA were regarded as conclusive. Albumin and C-reactive protein concetration in plasma were measured by turbidimetry using Cobas Mira Plus analyzer (Roche Diagnostics GmbH, Montclair, NJ, USA). Donor testing for serum antibodies against hepatitis A, C and E, HIV (antibodies and p24 antigen), Epstein-Barr virus, cytomegalovirus, *Treponema pallidum, Entamoeba histolytica, Toxoplasma gondii, Toxocara canis, cati and suis* and HBs antigen was performed by ELISA using Cobas Elecsys analyzer (Roche Diagnostics GmbH, Montclair, NJ, USA). Donor feces samples were cultivated on selective deyoxycholate-citrate agar to detect Salmonella species, on Skirrow Campylobacter selective agar to detect Campylobacter species and ChromID ESBL and ChromID CARBA (BioMèrieux, Lyon, France) to detect multidrug-resistant Gram-negative bacilli.

### Statistical analysis

Quantitative variables are expressed as medians and 25th and 75th percentiles. Normal distributions of quantitative variables were assessed using the Kolmogorov-Smirnov test. Medians of quantitative variables between single FMT success and single FMT failure groups were compared using the Mann-Whitney nonparametric test. Univariate analyses of associations of categorical variables were assessed using the Chi-square test. Multivariate analysis of associations of variables with the probability of single FMT failure was assessed using binary logistic regression. Categorical variables that were associated with single FMT failure with a probability of at least 10% (*p* <  0.1) and quantitative variables that differed with a significance of at least p <  0.1 were included in the multivariate analysis. For statistical analysis, we used SPSS version 20 (International Business Machines Corporation, Armonk, NY, USA).

## Results

A total of 60 patients that underwent single FMT (34 women, 26 men) were included in the study. Overall, 15 patients (25%) experienced single FMT failure. 24 patients (40%) had CCI ≥ 7, and 45.0% patients with CCI ≥ 7 experienced failure of single FMT.

72 patients admitted for recurrent CDI were screened. A total of 60 patients (34 women, 26 men) were recruited, underwent FMT and were included in the study. In all, 15 patients (7 women, 8 men) experienced single FMT failure, which means that single FMT failed in 25% of patients. A total of 27 patients (45%) underwent FMT after more than 1 previous recurrence. In all, 24 patients (40%) had CCI ≥ 7, and 12 (50%) of these patients experienced single FMT failure. Only 4 patients (6.66%) from the group of patients with CCI < 7 experienced FMT failure (Fig. 2). The basic characteristics of our cohort are presented in Tables 1 and 2. Patients with single FMT failure had a significantly higher CCI median and had a significantly lower albumin concentration (Table 1). There was no difference in age, CRP concentration, leukocyte count and time from FMT to first defecation. In univariate analysis, we found an association of FMT failure with CCI ≥ 5, CCI ≥ 7, CCI ≥ 9 and male sex. Because of the narrowest 95% confidence interval range for CCI ≥ 7 and FMT failure association in univariate analysis, we used CCI ≥ 7 as cut-off for definition of comorbid status for our study purposes. We find no association with concomitant PPI therapy, severe CDI, hospital-acquired CDI, history of ≥3 recurrences and CCI ≥ 3 (Table 3). In multivariate analysis, CCI ≥ 7 was positively associated with risk of FMT failure. Other variables included in the multivariate analysis (sex, age, time from FMT to first defecation, concomitant PPI therapy, severe CDI, albumin concentration and time from FMT to first defecation) were not associated with single FMT failure risk (Table 4).

**Table 1:** Basic characteristic, quantitative variables

	All patients (*n* = 60)	Single FMT failure (*n* = 15)	Single FMT success (*n* = 45)
Age (years)	73 (61–82)	75 (70–82)	72 (58–80)
CCI	6 (4–8)	7 (6–9)*	5 (3–6)
Leukocytes (1000/ml)	9.52 (5.96–14.08)	7.48 (5.52–12.05)	10.21 (6.59–15.46)
CRP (mg/l)	47.16 (7.24–110.25)	47.30 (5.62–111.00)	44.14 (6.54–119.50)
Albumin (mg/l)	32.25 (27.27–39.15)	28.90 (25.30–32.72)*	34.15 (26.77–39.15)
Time from FMT to first defecation	20 (12–24)	24 (3–24)	20 (12–24)

* Statistically significant difference between groups with single FMT failure and single FMT success (Mann-Whitney, *p* <  0.05)

Values are displayed as median (IQR); *CCI* Charlson Comorbidity Index, *CRP* C-reactive protein, *IQR* interquartile range, *FMT* faecal microbial transplantation, and n – number of subjects in cohort

**Table 2:** Basic characteristic, categorical variables

Variables	n = 60 (%)
Gender (male)	26 (43.33%)
Severe CDI	23 (38.33%)
Hospital-acquired infection	51 (85.00%)
History of 3 and more recurrences of CDI	11 (18.33%)
Diabetes mellitus type 2	13 (21.66%)
CCI ≥ 7	24 (40.00%)
Oncologic disease	11 (18.33%)
Dementia	10 (16.67%)
Chronic kidney disease	11 (18.33%)
Heart failure	22 (30.04%)
Liver disease, except cirrhosis	19 (31.67%)
Liver cirrhosis	2 (3.33%)
Myocardial infarction	10 (16.67%)
Arterial hypertension	43 (71.67%)
Stroke or TIA	10 (16.67%)
Hemiplegia	6 (10.00%)
Peptic ulcer	8 (13.33%)
Connective tissue disease	4 (6.67%)
COPD	9 (15.00%)
Peripheral artery disease	6 (10.00%)
AIDS	0 (0%)
HIV	1 (1.67%)
PPI therapy	25 (41.50%)

*AIDS* acquired immunodeficiency syndrome, *CCI* Charlson Comorbidity Index, *CDI Clostridium difficile* infection, *HIV* human immunodeficiency virus, *n* number of subjects in cohort, *PPI* proton pump inhibitor, and *TIA* transient ischemic attack

**Table 3:** Univariate analysis of categorical variables with the single FMT failure

Variables	RR	95% CI of RR	p
CCI ≥ 3	1.208	0.979–1.490	0.168
CCI ≥ 5	1.530	1.048–3.236	< 0.05
CCI ≥ 7	2.88	1.809–7.065	< 0.001
CCI ≥ 9	6.844	2.023–23.159	< 0.05
Sex (male)	0.528	0.275–0.821	< 0.05
Hospital-acquired infection	1.760	0.278–17.026	0.795
History of 3 and more recurrences	1.090	0.850–1.397	0.541
Concomitant PPI therapy	1.412	0.772–2.582	0.290
Severe CDI	1.600	0.935–2.737	1.157

*CCI* Charlson Comorbidity Index, *CI* confidence interval, *FMT* faecal microbial transplantation, *n* number of subjects in cohort, *p – probability* Binary logistic regression, *PPI* proton pump inhibitor, and *RR* relative risk

**Table 4:** Multivariate analysis (Binary logistic regression) of variables with single FMT failure

variable	OR	95% CI of OR	p
CCI ≥ 7	6.995	1.469–30.636	< 0.05
Age	0.984	0.927–1.046	0.607
Sex (male)	0.687	0.145–3.265	0.687
Time from FMT to first defecation	0.997	0.942–1.054	0.914
Albumin concentration	1.090	0.964–1.233	0.170

*CCI* Charlson Comorbidity Index, *CI* confidence interval, *FMT* faecal microbial transplantation, *n* number of subjects in cohort, *p – probability* Binary logistic regression, and *OR* odds ratio

## Discussion

### FMT failure and comorbid status

Our results suggest that comorbid status assessed using CCI and for study purposes defined as CCI ≥ 7 is positively associated with the single failure of FMT in the treatment of recurrent CDI. It indicates that comorbidity is a potent risk factor for FMT failure. CDI is the most common cause of health care-associated diarrhoea in Europe [4]. Tendency for recurrent infections makes treatment of CDI very challenging. More than 20% of patients with CDI experience the recurrence of disease within 2 months, and if CDI recurs, the chance for a subsequent recurrence increases to 60% [7]. FMT is a highly effective method to prevent recurrent CDI. However, it fails in about 5 to 20% of patients [15]. The mechanisms that lead to FMT failure and its risk factors are poorly understood. Risk factors of developing recurrent CDI after a first CDI episode treated with antibiotic therapy only are well-known. There is large body of evidence supporting the role of comorbid status as important risk factor of developing recurrent CDI after standard vancomycin therapy [8–12]. On the other hand, risk factors for the development of further recurrences after FMT are more or less unknown. To our knowledge, only 3 studies have tried to identify the risk factors of FMT in the treatment of recurrent CDI, and none of these studies conclusively linked comorbidity to the risk of FMT failure [17–19]. A relatively large retrospective study by Meighani et al. compared the risk of FMT failure between groups of patients with recurrent CDI stratified by the CCI. They defined comorbid status as CCI ≥ 3. However, they found no association of CCI ≥ 3 and risk of FMT failure [18]. Our patients were more comorbid than in the study by Meighani et al. In their study, less than 40% of all patients had CCI ≥ 3. In our study, more than 40% had CCI ≥ 7. It is possible that Meighani et al. were unable to find an association between CCI and FMT failure because their population was less comorbid or and/or they chose too low of a CCI cut-off to stratify their patients. Another 2 retrospective studies did not assess the association of FMT failure with CCI or any other direct surrogate marker of comorbidity. A study by Fischer at al. found that inpatient status during FMT is associated with the risk of FMT failure. They suggested that inpatient status during FMT is an indicator for worse overall health condition, which might be affected by the presence of serious comorbidities [19]. A study by Patron et al. did not look for any comorbidity markers of FMT failure [17]. In our study, patients that experienced FMT failure were older. However the difference was not statistically significant. Patron et al. and Fischer at al. found that age is associated with FMT failure [17, 19]. Advancing age has also been identified as a risk factor for developing recurrent CDI in patients treated with standard antibiotic therapy [24]. Age is also one of the variables included in the CCI equation [21, 22]. Naturally, age might be considered a possible confounder for association of FMT failure and comorbidity status. Therefore, we included age in the multivariate model and CCI ≥ 7 was nevertheless significantly associated with FMT failure. To objectively assess this association of comorbidity to the risk of FMT failure, we used CCI as a well-established surrogate marker of comorbidity. It was developed and validated by numerous studies as a measure of 1-year mortality risk and marker of disease burden. The CCI is used extensively in clinical research to address the influence of comorbidities on prognosis and outcome. Variables that affect CCI are age, presence of arterial hypertension, diabetes mellitus, solid tumors, leukaemia, lymphoma, heart failure, liver cirrhosis, hepatopathy, stroke or TIA, dementia, peptic ulcer disease, peripheral artery obliterating disease, myocardial infarction, connective tissue disease, COPD and AIDS [20, 21].

### FMT failure and albumin concentration

Patients with single FMT failure had significantly lower albumin concentrations at the time of diagnosis of recurrence. However, in the multivariate analysis, there was no association between albuminuria and single FMT failure. The association of hypoalbuminemia with FMT failure has not yet been studied. Hypoalbuminemia is known risk factor for developing recurrent CDI after treatment with standard antibiotic therapy [25]. A study by Cohen et al. concluded that albumin concentration is positively associated with CCI in patients with CDI but not with disease severity objectivized as the risk of death within 30 days of admission [26]. We suggest that comorbid status is the confounder of the association between albuminemia and single FMT success. Therefore, it is not associated with FMT failure if the analysis is controlled for CCI and age. Another explanation is the low number of subjects and therefore false negativity.

### FMT failure and sex

In the univariate analysis, female sex was positively associated with the risk of single FMT failure. However, the association disappeared in multivariate analysis. Meighani et al. found that female sex was associated with less favorable outcomes of FMT in univariate analysis. However, they suggested that the association of female sex and FMT failure was rather caused by confounding factors other than causality, since they did not control the analysis for possible confounders [18]. Fischer at al. found no association of FMT failure with sex in multivariate analysis [19] Patron et al. found no association of sex and risk of FMT failure even in univariate analysis [17].

### FMT failure and multiple recurrences of CDI

We failed to conclusively demonstrate that a history of multiple recurrences (defined as more than 2 previous recurrences) is the risk factor of single FMT failure. Studies by Patron et al. and Fischer at al. concluded that an increasing number of previous recurrences is positively associated with FMT failure [17, 19]. We suggest that the lack of significant association in our study has been caused by the fact that many of the patients in our cohort had FMT as a treatment for their first CDI recurrence (45%), and only 11 (18.33%) patients had more than 2 previous recurrences.

### FMT failure and PPI therapy

In our study, the risk of FMT failure was not associated with concomitant PPI therapy. PPI therapy is associated with the risk of recurrence after standard vancomycin therapy, possibly because it facilitates re-infection with *C. difficile* spores [27]. We suggest that the restitution of gastrointestinal flora diversity induced by FMT is enough to protect from *C. difficile* colonization and overgrowth [15]. The low number of patients in our study might also contribute to the lack of association between FMT failure and concomitant PPI therapy.

### Other predictors of FMT failure

Previously mentioned studies found several other factors that might possibly affect the success rate of FMT. Meighani et al. suggested that hospital-acquired CDI is a risk factor for FMT failure [18]. 85% of our patients had hospital-acquired CDI, and we found no association between inpatient status and FMT failure. However, due to the low number of cases of community-acquired CDI in our cohort, we were unable to make any conclusions due to the high probability of false negativity. We also did not find any association of FMT failure with severe CDI, which is also in contradiction with the results of Fischer et al. [19]. Fischer et al. also concluded that inpatient status during FMT was a risk factor for FMT failure [19]. As FMT in our department is done as an inpatient procedure, all our patients were treated as inpatients during FMT. Therefore, our results are not fully comparable. Finally, a study by Patron et al. found that signs of colitis on abdominal CT scan and presence of inflammatory bowel disease (IBD) are associated with FMT failure [17]. We were unable to assess the effect of the presence of IBD, because our study did not include patients with IBD. We could not study the effects of colitis visible on CT scan on the risk of FMT failure, because we did not perform abdominal CT scans during the recurrence of CDI. Parlor et al. also found that the risk of FMT failure was significantly lower if a vancomycin taper rather than 10 days of oral vancomycin preceded the FMT procedure [17]. We could not study the effects of vancomycin taper, as all our patients were treated with 10 days of oral vancomycin followed by FMT.

### Study advantages

Our study has a prospective design. Studies that focus on predictors of FMT failure conducted to this date have had a retrospective design. Therefore, they might suffer from certain flaws inherited in the design of most retrospective studies. Their populations were heterogeneous in the means of FMT realization. Methods of FMT vary by stool type (fresh or frozen) and method or location of delivery (NG tube, sigmoidoscopy, colonoscopy, or rectal retention enema). In our study, we used only fresh stool samples delivered by rectal retention enema. However, we can only speculate if these differences between studies might be responsible for the discrepancies in the results.

### Study limitations

Our study has several limitations. Above all, the relatively low number of patients led to an inability to conclusively assess associations between FMT failure and other possible risk factors (age, time from FMT to first defecation, concomitant PPI therapy, severe CDI, and hospital- acquired infection) because of the high probability of false negativity. We followed our patients only for 12 weeks after FMT, so possible recurrence past this point might be missed. However, a large study by Fischer at al. found that more than 97% of all failures happen in first month after FMT [19]. Therefore, we suggest that a 12 week follow-up period is sufficiently long. Our patients were also instructed to call investigators if any signs of possible recurrence occurred in the first 6 months after their last follow-up visit, and no cases of recurrence were recorded. We did not perform ribotyping of *C. difficile* strains in our recipients, so we were unable to exclude the effects of infection by hyper-virulent strains as a possible confounder. We were also unable to distinguish between re-infection by a new strain and relapse caused by the original strain in patients experiencing FMT failure.

### Efficacy of single FMT by rectal enema in our population

In all, 25% of our patients experienced single FMT failure, and the efficacy of single FMT in our study was 75%, which seems to be much less than the 95% found in a large meta-analysis by Quraishi et al. However, in the mentioned meta-analysis, they evaluated overall efficacy, including patients with multiple FMTs who had undergone re-transplantation after they experienced single FMT failure [15]. For example, Fischer et al. also used single FMT failure as a primary endpoint, and the efficacy was approximately 79% [19]. Another explanation is that our cohort included a high proportion of elderly comorbid patients, and if comorbidity is a risk factor for FMT failure, lower efficacy is expected. The efficacy of FMT in the elderly population is known to be much lower (69–83%) [28, 29].

### Practical applications

Application of identification of manageable risk factors, such as concomitant PPI therapy during FMT may lead to improved methods of FMT. The practical application of identification of unmanageable risk factors for single FMT failure may lead to the development of a model predicting single FMT failure. In patients with high risk for single FMT failure, alternative methods of recurrent CDI prevention, such as multiple FMT or FMT after vancomycin taper, might be developed as standard therapy. In our study, 45.83% patients with CCI ≥ 7 experienced single FMT failure in the inpatient setting. This failure rate seems to be unacceptably high, which underlines the importance of improving the FMT protocol in elderly comorbid patients.

## Conclusions

We concluded that comorbid status surrogated by CCI is positively associated with FMT failure in the treatment of recurrent CDI. This association is independent of age, sex and markers of severity of disease (CRP, leukocyte count). That indicates that comorbid status is potent risk factor for FMT failure.

## Data Availability

The dataset used and analysed during this study is available from the corresponding author upon request.

## Abbreviations

AIDS: Acquired immunodeficiency syndrome
ATB: Antibiotic
CCI: Charlson Comorbidity Index
CDI: *Clostridioides difficile* infection
COPD: Chronic obstructive pulmonary disease
CRP: C-reactive protein
ELISA: Enzyme-linked immunosorbent assay
FMT: Faecal microbial transplantation
HIV: Human immunodeficiency virus
IQR: Interquartile range
MI: Myocardial infarction
PPI: Proton pump inhibitor
TIA: Transient ischemic attack
